# Aqueous-Based Coaxial Electrospinning of Genetically Engineered Silk Elastin Core-Shell Nanofibers

**DOI:** 10.3390/ma9040221

**Published:** 2016-03-23

**Authors:** Jingxin Zhu, Wenwen Huang, Qiang Zhang, Shengjie Ling, Ying Chen, David L. Kaplan

**Affiliations:** 1 College of Materials Science and Engineering, Taiyuan University of Technology, 79 West Yingze Street, Taiyuan 030024, China; zhujingxin@tyut.edu.cn; 2 Department of Biomedical Engineering, Tufts University, 4 Colby Street, Medford, MA 02155, USA; wenwen.huang@tufts.edu (W.H.); qiang.zhang@wtu.edu.cn (Q.Z.); shengjie@mit.edu (S.L.); ying.chen@tufts.edu (Y.C.); 3 School of Textile Science and Engineering, Wuhan Textile University, Wuhan 430073, China; 4 Laboratory for Atomistic and Molecular Mechanics (LAMM), Department of Civil and Environmental Engineering, Massachusetts Institute of Technology, Cambridge, MA 02139, USA

**Keywords:** coaxial electrospinning, core-shell structure, silk-elastin-like protein polymer, silk fibroin

## Abstract

A nanofabrication method for the production of flexible core-shell structured silk elastin nanofibers is presented, based on an all-aqueous coaxial electrospinning process. In this process, silk fibroin (SF) and silk-elastin-like protein polymer (SELP), both in aqueous solution, with high and low viscosity, respectively, were used as the inner (core) and outer (shell) layers of the nanofibers. The electrospinnable SF core solution served as a spinning aid for the nonelectrospinnable SELP shell solution. Uniform nanofibers with average diameter from 301 ± 108 nm to 408 ± 150 nm were obtained through adjusting the processing parameters. The core-shell structures of the nanofibers were confirmed by fluorescence and electron microscopy. In order to modulate the mechanical properties and provide stability in water, the as-spun SF-SELP nanofiber mats were treated with methanol vapor to induce β-sheet physical crosslinks. FTIR confirmed the conversion of the secondary structure from a random coil to β-sheets after the methanol treatment. Tensile tests of SF-SELP core-shell structured nanofibers showed good flexibility with elongation at break of 5.20% ± 0.57%, compared with SF nanofibers with an elongation at break of 1.38% ± 0.22%. The SF-SELP core-shell structured nanofibers should provide useful options to explore in the field of biomaterials due to the improved flexibility of the fibrous mats and the presence of a dynamic SELP layer on the outer surface.

## 1. Introduction

Electrospinning is a relatively simple and effective method for the production of uniform, continuous fibers with diameters ranging from the nanoscale to the microscale. These micro/nanofibers in the form of nonwoven mats have been used widely in various biotechnologies, such as wound healing, tissue engineering, and drug delivery [1,2]. Silk fibroin (SF), derived from *Bombyx mori* cocoons, is a protein polymer which has been extensively studied and used for biomaterial applications [3,4,5,6]. SF is non-immunogenic and can be fabricated into nanofibers by electrospinning from aqueous processing methods, either as a blend with poly(ethylene oxide) PEO [7] or from high concentration SF aqueous solutions [8]. However, the breaking elongation of these mats tends to be low and the mats are generally brittle. There is a need for silk - based biomaterials with improved mechanical properties for biomedical-related uses, as well as systems that provide more dynamic functions.

Silk-elastin-like protein polymers (SELPs) are genetically-engineered block copolymers consisting of silk-like (GAGAGS) and elastin-like (GVGVP) repeating units in defined ratios. SELPs are stimuli sensitive materials (e.g., pH, temperature, ionic strength) [9,10,11] and exhibit interesting biomechanical properties, suggesting potential utility in drug delivery systems and tissue engineering applications [12,13]. Moreover, SELPs can be processed in aqueous conditions, and have been explored in various forms, such as nanoparticles [14], hydrogels [15], films [16] and fibers [17]. SELPs can also be electrospun into fiber mats. However, electrospinning of SELPs is challenging due to the low viscosity aqueous solutions. The addition of a polyethylene glycol-sodium dodecyl sulfate (PEO-SDS) complex in SELP aqueous solution were used to increase the viscosity, and nanofibers of a SELP, named SELP-67K, were obtained by electrospinning [18]. Nanoribbons with diameters of 25 and 1800 nm were generated from a SELP-47K aqueous solution [19]. Biocompatible tissue scaffolds were also generated by electrospinning a solution of 15 wt % SELP-47K in formic acid [20]. The electrospinning of SELP-1020-A and SELP-59A from formic acid and aqueous solution has also been recently described [21]. These electrospun fiber mats showed no cytotoxicity and supported adhesion and proliferation of human skin fibroblasts. While these studies demonstrated the potential of using SELP to produce nanofibers, most of these mats still contained chemicals or solvents after electrospinning which required removal prior to further utility. Moreover, the SELPs are generated via biotechnology processes and thus costs per amount are relatively high; options to utilize their properties but minimize the amounts needed would thus be very attractive.

In order to gain the benefits of both SF and SELPs, here we report a method to generate SF-SELP core-shell nanofibers from aqueous solutions by coaxial eletrospinning. Theoretical and experimental studies on coaxial electrospinning suggest that this research area has significantly advanced nanofabrication techniques and has potential for biomedical applications, such as for tissue engineering scaffolds, drug release systems, wound dressings, and related topics [2,22,23,24,25,26,27]. In conventional coaxial electrospinning, the shell solution acts as a template to the core material and imparts shear stress to overcome the interfacial tension between the two solutions via viscous drag and contact friction. Therefore, high viscosity shell solutions are desirable for conventional coaxial electrospinning. In previous studies [22,23,24,25,26], the shell fluid in general has had good electrospinnability and the core solution can have either poor or good electrospinnability, so that the shell solution can serve as a template and be removed at later stage if needed. For example, silk nanofibers were prepared by coaxial electrospinning, in which PEO served as a template shell for the silk core and was then leached out with water to produce silk nanofibers [27].

In the present work, the objective was to produce SF-SELP core-shell structured nanofibers by using the core solution as a spinning aid to electrospin nonelectrospinnable shell solutions. This method would serve as a roadmap for the fabrication of functional nanofibers using nonelectrospinnable materials, such as genetically designed functional peptides. To demonstrate this concept, SF was chosen as a core material because of its mechanical strength and good electrospinnability, while SELP was used as the shell due to its elasticity and stimuli sensitive features; towards responsive and dynamic shell systems on the fibers. In order to determine the most suitable concentration of the core and shell solutions, the rheological behavior of the SF and SELP aqueous solutions with different concentrations were evaluated. In addition, the effects of coaxial electrospinning process parameters, such as the flow rate of the core and shell solutions, the applied voltage, and the resultant fiber diameters were investigated.

## 2. Experimental Section

### 2.1. SELP (S2E8Y) Solution Preparation

The SELP, named S2E8Y, was genetically designed to have a silk to elastin block ratio of 1:4, and molecular weight of 53 kDa, where S is the silk block with sequence GAGAGS and E is the elastin block with sequence GYGVP. The expression plasmids were constructed using our previously established procedures [11] and expressed under the T7 promoter in *E. coli* strain BL21Star (DE3) (Invitrogen, Carlsbad, CA, USA) in a New Brunswick BioFlo 3000 bioreactor (New Brunswick Scientific, Edison, NJ, USA). The protein was purified by the inverse transition cycling (ITC) method, as described previously [9,10]. The purity of the proteins was monitored via sodium dodecyl sulfate polyacrylamide gel electrophoresis (SDS-PAGE). To prepare S2E8Y solution for electrospinning, lyophilized S2E8Y powder was dissolved in deionized water at 12, 13.5 or 15 wt %. The solutions were maintained at 4 °C overnight and set at room temperature for 30 min before electrospinning.

### 2.2. Silk Fibroin (SF) Solution Preparation

Silk fibroin (SF) solution was prepared from *B. mori* cocoons as described previously [28]. Briefly, cocoons were boiled twice for 30 min in a solution of Na_2_CO_3_ (0.02 M), rinsed and then dried at ambient conditions overnight. The dried degumming fibers were solubilized in a 9.3 M LiBr solution at 60 °C for 4 h, yielding a 20% (*w*/*v*) solution. Silk solutions were dialyzed against deionized water using dialysis tubing (MWCO 3.5 kDa) for 72 h to remove salts. SF solution was concentrated by airflow in Slide-a-Lyzer dialysis cassettes (Thermo Scientific, Waltham, MA, USA) at 10 °C to produce a 20 wt % SF solution, then the pH of the solution was adjusted to 6.0 by adding a 0.1 M 2-(N-morpholino) ethanesulfonic acid (MES)-trishydroxymetnyl aminomethane (Tris) buffer solution into a 20 wt % solution in a volume ratio of 1:2. SF solution was concentrated again by airflow in a 50 mL beaker at 10 °C to 26–29 wt %. The concentration of the solution was monitored by weighing the remaining solid after drying.

### 2.3. Rheology

Rheological analysis of SF and S2E8Y solutions was performed on an ARES-LS2 rheometer (TA Instruments, New Castle, DE, USA) using a 25 mm cone and plate geometry (angle: 0.0994 rad, gap set to 0.5 mm) at 25 °C. The viscosities of SF and S2E8Y aqueous solutions with different concentrations under shear were investigated through a logarithmic steady shear rate from 0.01 to 1000 s^−1^.

### 2.4. Coaxial Electrospinning

Coaxial electrospinning was performed using a vertical setup containing a variable high DC voltage power supply (Gamma High Voltage Research ES-30P, Ormond Beach, FL, USA) and two individual syringe pumps (Thermo Scientific). The coaxial spinneret (Ramé-hart Instrument Co., Succasunna, NJ, USA) was comprised of a 20G (inside diameter (i.d.) 0.584 mm, outside diameter (o.d.) 0.889 mm) inner needle concentrically mounted on a 12G (i.d. 2.16 mm, o.d. 2.77 mm) outer needle. SF and S2E8Y solutions were poured into 5 and 3 mL plastic syringes which were connected to the syringe pumps and the coaxial spinneret. The coaxial spinneret was connected to the high voltage power supply. The flow rates of the core (SF solution) and shell (S2E8Y solution) were controlled by two separate pumps. The electrospun fibers were collected on an aluminum foil wrapped 10 cm-diameter grounded metal plate. The applied voltage was varied in the range of 20–30 kV, and the distance between the tip and the collector was 10 or 12 cm, respectively. All the electrospinning processes were carried out at around 23 ± 3 °C and 45% ± 5% humidity.

### 2.5. Standard Electrospinning

SF fiber mats were produced by the same vertical setup using a single needle 16G (i.d. 1.19 mm) to serve as a control sample. The process parameters were set as following: the concentration of SF was 29 wt %; the flow rate of solution was 0.8 mL/h; applied voltage was 25 kV; the distance between the tip and the collector was 12 cm; and electrospinning processes were carried out at around 23 ± 3 °C and 45% ± 5% humidity.

### 2.6. Morphology of the Electrospun SF-SELP Fiber Mats

Morphology of the electrospun SF-SELPs fiber mats was observed with a field emission scanning electron microscope (FE-SEM) (Ultra55, Zeiss, Oberkochen, Germany). Samples were sputter-coated with gold for 60 s for improved conductivity. The SEM images were taken at 5 kV. The average diameter of the fibers in each specimen was generated from 100 randomly selected fibers on images and expressed as mean ± standard deviation (SD). All measurements and statistics were handled by Image J. software (National Institutes of Health, Bethesda, MD, USA).

### 2.7. Core-Shell Structure Characterization

To visualize the core and shell phases, 5 μg/mL rhodamine-B (Merck Chemicals, Darmstadt, Germany) and 40 μg/mL 70 kDa fluorescein isothiocyanate-dextran (Sigma-Aldrich, Inc. St. Louis, MO, USA) were added to the 29 wt % SF solution and 12 wt % S2E8Y solution, respectively, and coaxially electrospun as described previously. The nanofibers were directly deposited on the glass coverslips and imaged under phase contrast and fluorescence modes using a BZ-X700 fluorescence microscope (Keyence Co., Itasca, IL, USA). Wide-green and wide-blue filter cubes were used to detect the rhodamine B-stained core and FITC-dextraned-stained shell phases, respectively [29]. The core-shell structure was further characterized using a JEM-1011 transmission electron microscope (TEM) (JEOL Ltd., Tokyo, Japan ) operated at 80 kV. The electrospun nanofiber samples for the TEM observation were prepared by directly depositing the as-spun fibers onto copper grids.

### 2.8. Fourier Transform Infrared Spectroscopy (FTIR)

FTIR analysis was performed using an FT/IR-6200 Spectrometer (Jasco, Easton, MD, USA), equipped with a triglycine sulfate detector in attenuated total reflection (ATR) mode. The absorbance spectrum for each sample was obtained by averaging 64 scans with a resolution of 2 cm^−1^ over the wavenumber range from 600 to 4000 cm^−1^. The background spectra were collected under the same conditions and subtracted from the scan for each sample.

### 2.9. Methanol Treatment

The as-spun electrospun fiber mats were treated with methanol vapor in a vacuum desiccator for 72 h. During treatment, a Petri dish containing 200 mL MeOH was placed in the bottom chamber of a sealed desiccator, while the SF and SF-SELP fiber mats with aluminum foil were placed on a ceramic desiccator plate in the top chamber. Treated nanofiber mats were then peeled off the aluminum foil for FTIR and mechanical analysis.

### 2.10. Tensile Tests of Electrospun Mats

Specimens were prepared for mechanical testing following previous methods [30,31]. Fiber mats were cut into 40 mm × 10 mm rectangular samples, and mounted on a cardboard window frame (inside: 20 mm× 20 mm, outside: 40 mm × 40 mm) for testing. Sample thickness was measured with a digital micrometer (Mitutoyo, Aurora, IL, USA). Tensile tests were performed using an Instron 3366 testing instrument (Instron Co., Norwood, MA, USA) equipped with a 100 N load cell at a rate of 1 mm·min^−1^ with gauge length of 20 mm. The stress strain data were obtained automatically. Young’s modulus was calculated by the slope of each stress-strain curve in its elastic deformation region [32]. Data was collected from 10 measurements for each specimen and expressed as mean ± standard deviation (SD).

## 3. Results and Discussion

### 3.1. Protein Characterization

**Figure 1:**
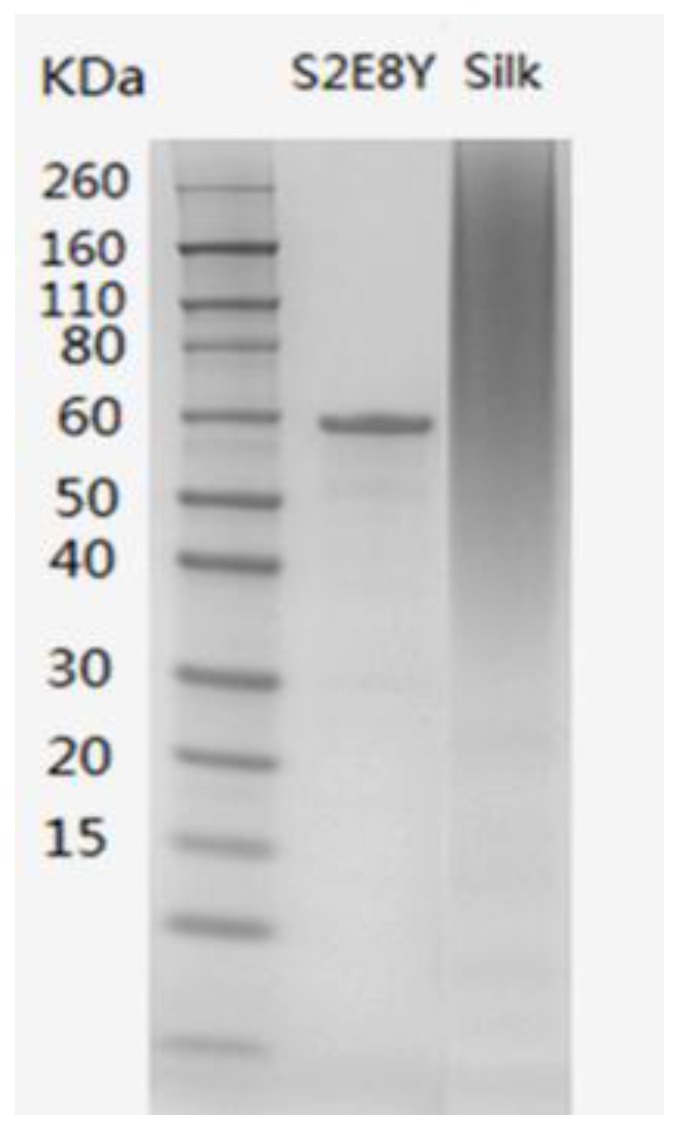
Sodium dodecyl sulfate polyacrylamide gel electrophoresis (SDS-PAGE) of purified S2E8Y and regenerated silk fibroin (SF).

An SDS-PAGE gel for the purified S2E8Y and regenerated SF is shown in Figure 1. The S2E8Y exhibited a single band suggesting the high purity of purified recombinant protein. The apparent molecular weight is about 60 kDa, higher than the calculated molecular weight of S2E8Y, 53 kDa. The likely reasons for this discrepancy can be attribute to two factors: (1) a lower than normal amount of bound SDS on the SELP; and (2) molecular size and conformation that reduces protein mobility. The molecular weight of S2E8Y was therefore confirmed by MALDI-TOF. The volumetric productivity of S2E8Y was about 1250 mg/L. The regenerated SF exhibited a smear as a heterogeneous mixture with molecular weights from 40 to 260 kDa, as we have previously reported [33].

### 3.2. Rheological Behavior of Solutions

**Figure 2:**
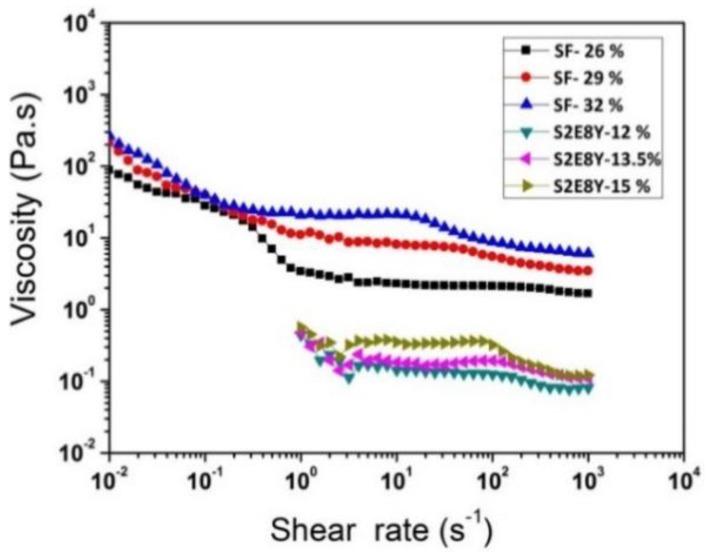
Rheology of SF and S2E8Y solutions with different concentrations.

Rheological curves of SF and S2E8Y solutions with different concentrations are shown in Figure 2. Flow curves of SF and S2E8Y solutions exhibited the same trends in the range of concentrations investigated. Viscosity-dependent shear rate showed greater shear thinning at lower shear rate, Newtonian flow at intermediate shear rate, and lower shear thinning at higher shear rates. In the lower shear rate region, flow curves of SF solution showed greater shear thinning due to the destruction of weak intermolecular interactions in SF solution in the static state [34]. In the intermediate shear rate range, flow curves of SF solution showed a plateau viscosity region, termed the zero shear viscosity (η_0_), which suggested the formation of replacement interaction in a dynamic equilibrium [35]. As the concentration of SF increased, a shear thinning region emerged at higher shear rate, which was similar to pseudoplastic flow behavior of polymer solutions. These results implied that the shear-sensitivity of the solutions was enhanced by the increase in SF concentration, and shear thinning behavior was induced by the rearrangement and orientation of macromolecular chains along the shear direction [36]. Compared to the zero shear viscosity (about 0.2 Pa·s) of S2E8Y solutions, the zero shear viscosity of SF solutions (about 2–20 Pa·s) was higher than S2E8Y by one to two orders of magnitude. In general, the viscosity of SF and S2E8Y solutions increased with concentration, and compared to the viscosity of S2E8Y solutions, the viscosity of SF solution increased more significantly when the solution concentration was increased.

### 3.3. Effect of Processing on Electrospun SF-SELP Fibers

In most coaxial electrospinning, the shell polymer solution has sufficient viscoelasticity and good electrospinnability, and the core solution can have either poor or good electrospinnability [37,38]. In the present study in contrast to these prior studies, the SELP solutions with a relatively low viscosity were chosen as the shell, and the SF solutions with a relative high viscosity were chosen as the core. The process parameters such as concentration, flow rate, distance between the needle tip and collector, and applied voltage were studied in order to ensure a stable state cone-jet for the formation of homogeneous core-shell fibers.

**Figure 3:**
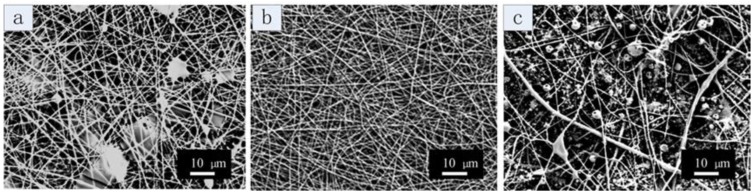
Scanning electron microscopy (SEM) images of coaxial electrospun SF-SELP nanofibers with various SF concentrations: (**a**) 26 wt % (**b**) 29 wt % (**c**) 32 wt %. The concentration of the shell SELP solution was fixed at 15%. The inner flow rate was 0.4 mL/h, the outer flow rate 0.17 mL/h, the tip to collector distance 12 cm and the applied voltage 20 kV.

The effect of SF concentrations on nanofiber morphology is shown in Figure 3. Other process parameters and fiber diameters are listed in Table 1. During electrospinning, the concentration of SF was varied from 26% to 32%, while the concentration of SELP solution was kept constant at 15 wt %. The 26% to 32% range was selected because the viscosity of SF solutions with concentrations lower than 26 wt % were too low to be electrospun and SF solutions with concentrations higher than 32% were not easy to electrospun due to the high viscosity. Other process parameters were kept the same for direct comparisons: the inner flow rate was 0.4 mL/h; the outer flow rate was 0.17 mL/h; the applied voltage was 20 kV; the tip to collector distance was 12 cm. Core solution at concentrations of 26 wt % and 32 wt % resulted in fiber mats with tiny droplets and beads on strings (Figure 3a,c). When the concentration of SF was 29 wt %, consistent fibers without beads were obtained, and the average diameter was about 301 ± 108 nm (Figure 3b). These results indicated that the concentration of the core solution, SF solution, is critical for the coaxial electrospinning. If the concentration was too low, e.g., 26 wt %, the viscosity of the solution was low (about 2.0 Pa·s), so that the core solution was not viscose enough to serve as a template for the shell solution to form the steady state of cone-jet. However, if the concentration was too high, e.g., 32 wt %, the applied electricity was not adequate to overcome the surface tension of Taylor cone and split the cone-jet flow into thin fibers due to the high viscosity (about 20 Pa·s) of the core SF solution [39].

**Figure 4:**
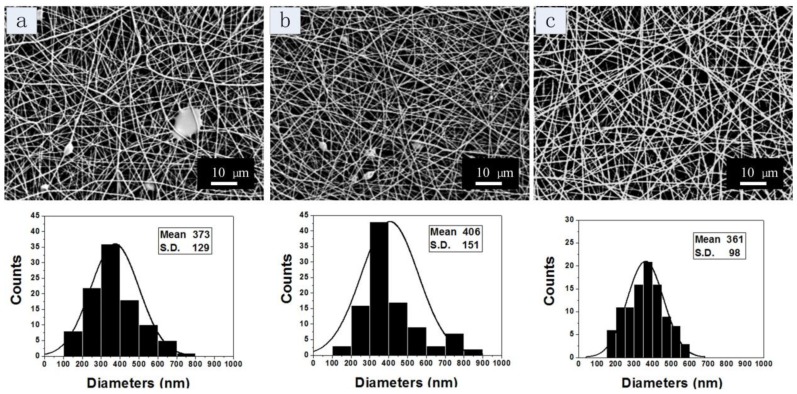
SEM images of coaxial electrospun SF-SELP nanofibers with various SELP concentrations: (**a**) 12 wt % (**b**) 13.5 wt % (**c**) 15 wt %. The concentration of SF solution was 29%. The inner flow rate was 0.4 mL/h, the outer flow rate 0.17 mL/h, the tip to collector distance 10 cm and the applied voltage 20 kV.

The effect of SELP concentration on the nanofiber morphology is shown in Figure 4. Other operating parameters are listed in Table 1. During electrospinning, the concentration of the SELP solution was increased from 12 to 15 wt %, while the concentration of the SF solution was kept constant at 29 wt %. When the SELP concentration was below 12 wt %, no continuous core-shell fiber was obtained, so the SELP concentration was selected between 12% and 15% to explore the lower concentration limits. For 12 wt % SELP solution, tiny droplets and beaded fibers formed in the fibers and the fiber average diameter was about 373 ± 129 nm (Figure 4a). For the 13.5 wt % SELP solution, beads were still visible but fewer in numbers and the fibers average diameter was about 406 ± 151 nm. When the concentration of SELP was increased to 15 wt %, uniform nanofibers with an average diameter of 361 ± 98 nm without beads were obtained. It indicated that increasing the concentration of SELP in solution promoted the formation of core-shell structured fibers, due to the improved viscosity and electrospinnability of SELP solution.

In summary, the SF concentration had a major impact on fiber morphology in the coaxial electrospining process. The SF solution with appropriate viscosity was able to serve as a spinning aid to successfully prepare nanofibers from low viscosity SELP solutions.

In order to generate a good “compound Taylor cone” with a core-shell structure, controlled and balanced injection speeds of the inner and outer fluids are critical to keep the “compound Taylor cone” in dynamic stabilization [40]. In the present study, 29 wt % electrospinnable SF solution was chosen as the core solution, which had shown good electrospinnable features based on Table 1, and 12 wt % nonelectrospinnable S2E8Y solution was chosen as the shell solution to demonstrate that the electrospinnable core solution played a critical role as a spinning aid to electrospin the nonelectrospinnable shell solution. The influence of the flow rate and applied voltage on the morphology and diameter of the core-shell fibers were also investigated. The process parameters and resultant fiber diameters are listed in Table 2.

**Figure 5:**
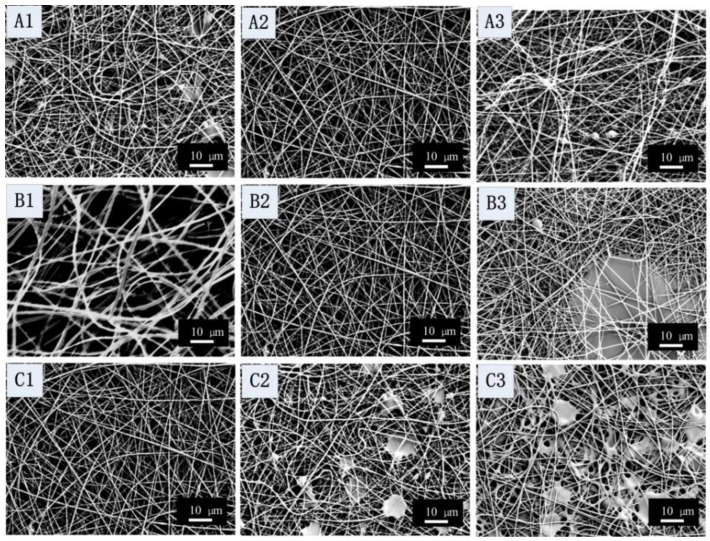
SEM images of coaxial electrospun SF-SELP nanofibers at different processes parameters: (**A1**, **A2**, **A3**) inner flow rates at 0.2, 0.4, and 0.6 mL/h, respectively; (**B1**, **B2**, **B3**) outer flow rates at 0.07, 0.17, and 0.34 mL/h, respectively; (**C1**, **C2**, **C3**) applied voltage at 20, 25, 30 kV, respectively. Other processing parameters are listed in Table 2.

The influence of the core SF flow rate on the morphology and diameter of the fibers is shown on the top-line of Figure 5. For the 0.4 mL/h (Figure 5(A1)) and the 0.8 mL/h (Figure 5(A3)) conditions, the collected fiber mats had droplets and breads-on-strings. With the core SF flow rate was increased from 0.4 to 0.6 then to 0.8 mL/h, the average diameter of the nanofibers increased from 370 ± 121, to 408 ± 150 then to 474 ± 162 nm, respectively. These data show that the effect of the core solution flow rate in coaxial electrospinning is similar to that for single electrospinning. An increase in solution flow rate generally resulted in larger fiber diameters [41].

The middle line of Figure 5 shows the influence of the shell SELP flow rate on the morphology and diameter of fibers. When the shell SELP flow rate increased from 0.07 (Figure 5(B1)) to 0.17 mL/h (Figure 5(B2)), the average diameter of the nanofibers decreased from 712 ± 215 nm to 408 ± 121 nm because the shell solution provided solvent to “wet” the high viscosity core solution that resulted in thinner fibers. However, the morphology of the fiber mats was changed to larger droplets and beaded fibers when the flow rate of the SELP solution was 0.34 mL/h (Figure 5(B3)). This phenomenon can be attributed to the oversupply of the SELP shell solution.

The last line of Figure 5 shows the effect of applied voltage on the morphology and diameter of fibers. There were no breads on fibers at 20 kV (Figure 5 C1). With the applied voltage increased from 25 (Figure 5(C2)) to 30 kV (Figure 5(C3)), the amount of beads and droplets increased. This is because the strength of the electric field exceeded that required for the material, so that the lower viscosity shell solution could not follow the core fluid path and disassociated into separate paths [22].

**Figure 6:**
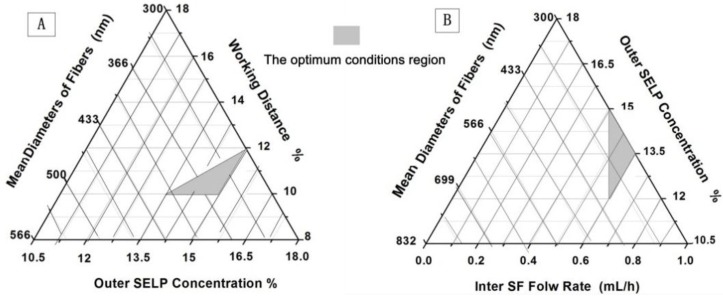
Conditions where to electrospin uniform SF-SELP nanofibers were generated: (**A**) mean diameters of fibers (nm) with outer SELP concentration (%) and working distance (cm); (**B**) mean diameters of fibers with inner flow rates (mL/h) and outer SELP concentration (%).

The results suggested that in this coaxial electrospinning process, the SF solution served as a spinning aid to successfully prepare nanofibers from nearly nonelectrospinnable SELP solution. The core SF solution carried the shell SELP solution through the formation of a stable Taylor cone and continuous jet ejection during the process. The optimum conditions to electrospin uniform SF-SELP nanofibers with different processes parameters are shown phase diagram in Figure 6. In addition, a combination of the following parameters was also critical. (a) Using the same solvent, water, led to low interfacial tension between the two solutions and maintained the steady and continuous jet by “wetting” the high viscosity core solutions [42]; (b) The higher conductivity of the shell layer helped the high shear stress to be applied on the core material, stabilized the coelectrospinning process and subsequent elongational force, and resulted in a thinner core; (c) Low vapor pressure of the solvent (boiling point of water = 100 °C) facilitated the spinning process. High vapor pressure solvents can produce unstable Taylor cones in coaxial electrospinning [43,44].

### 3.4. Core/Shell Structure of the Electrospun Fibers

**Figure 7:**
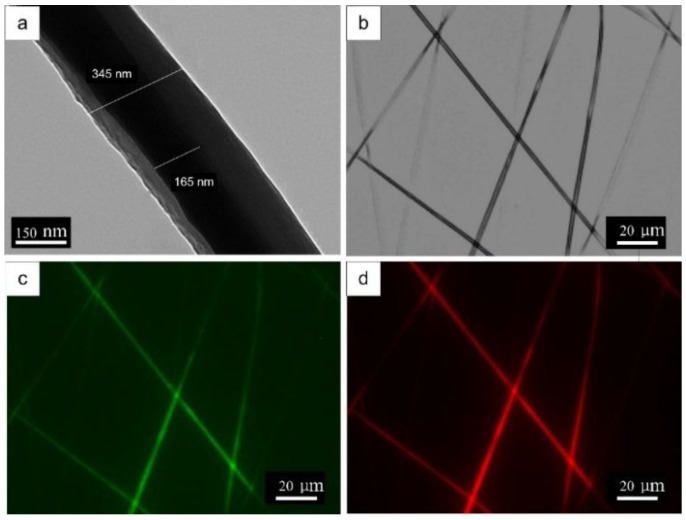
Core-shell structures of the SF-SELP nanofibers: (**a**) transmission electron microscopy (TEM) image and (**b**) phase-contrast image of fibers; (**c**) fluorescent image showing fluorescein isothiocyanate (FTIC)–dextran stained S2E8Y shell phase; and (**d**) fluorescent image showing rhodamine B stained SF cores phase.

TEM imaging were used to confirm the formation of SF-SELP core-shell structured fibers (Figure 7). Sharp boundaries of the SF core phase and SELP shell phase parallel to the fiber axis can be seen in Figure 7a. The bi-component nanofibers had an overall diameter of 345 nm with a core diameter of 165 nm. In addition, thin nanofiber mats with a fluorescein isothiocyanate (FTIC)–dextran labeled SELP shell phase and a rhodamine B labeled SF core phase were used to visualize the incorporation of the core and shell phases in individual fibers (Figure 7b–d). The green fluoresce from FITC-dextran (Figure 7c) and red fluoresce from rhodamine B (Figure 7d) suggested the SELP shell phase and SF core phase co-exist in the SF-SELP fibers.

### 3.5. Post-Treatment with Methanol and Structure of the Nanofiber Mats

**Figure 8:**
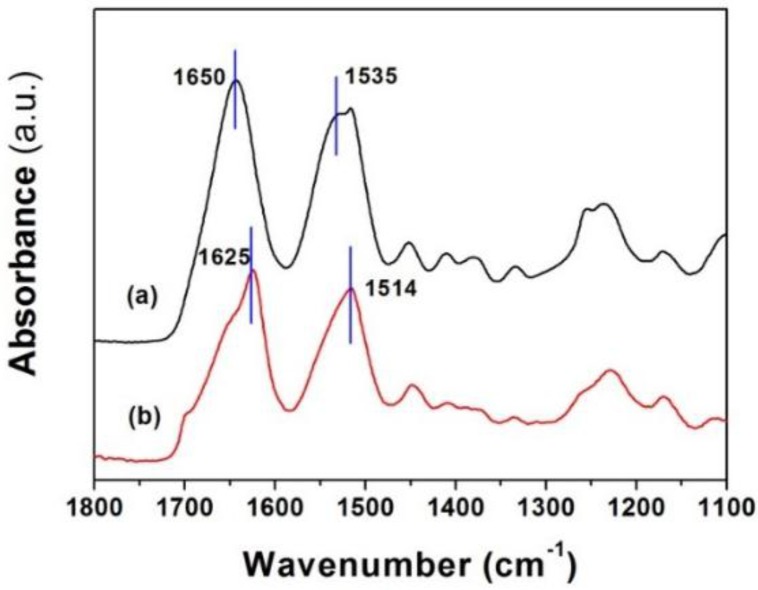
Fourier transform infrared spectroscopy (FTIR) spectra for SF-SELP core-shell fiber mats before and after methanol vapor treatment: (**a**) before methanol vapor treatment; and (**b**) after methanol vapor treatment.

The as-spun core-shell fiber mats are soluble in water. In order to extend their utility where contact with aqueous environments will be present, there is a need to stabilize the structure. To make fiber mats insoluble in water, methanol vapor was used to induce β-sheet formation [20]. ATR-FTIR was used to characterize the secondary structural changes induced by methanol in the electrospun mats. After methanol treatment, the amide I and amide II peaks shifted to lower wavenumbers (Figure 8). The initial peak centered at 1650 and at 1535 cm^−1^ (attributed to a random coil conformation [45,46]), shifted to values of 1625 and 1524 cm^−1^ (attributed to antiparallel beta-sheet structures [45,47]), respectively, reflecting a conversion from the unordered random coil structure to ordered β-sheets.

### 3.6. Mechanical Testing of SF and SF-SELP Fibers Mats Before and After Methanol Treatment

**Figure 9:**
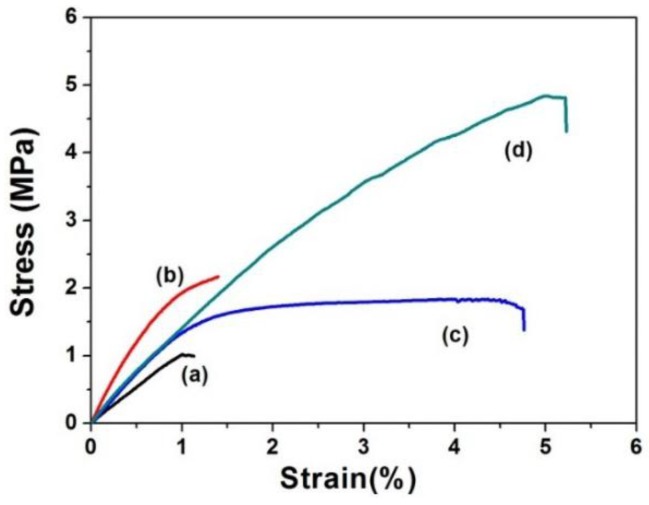
Stress-strain curves of SF fibers mats and SF-SELP core-shell fibers mats before and after methanol treatment: (**a**) SF fibers mats; (**b**) SF fibers mats after methanol treatment; (**c**) SF-SELPs core-sell fiber mats; and (**d**) SF-SELPs core-sell fiber mats after methanol treatment.

The tensile stress-strain curves of SF fibers mats and SF-SELP core-shell fiber mats before and after methanol treatment are shown in Figure 9. The average thickness, Young’s modulus, tensile strength and elongation at break are summarized in Table 3. Before methanol treatment, SF mats (Figure 9 curve a) had lower Young’s modulus and tensile strength which were 1.02 ± 0.12 MPa and 1.00 ± 0.22 MPa, respectively, meanwhile, the mats were brittle and easy to fracture with an elongation at break at only 1.05% ± 0.15%. In contrast, the SF-SELP mats (Figure 9 curve c) exhibited slightly higher Young’s modulus and tensile strength 1.30 ± 0.35 MPa and 1.72 ± 0.24 MPa, and good flexibility with elongation at break of 4.70% ± 0.31%. This difference may be attributed to the smaller diameters of SF-SELP core-shell fibers compared with the SF fibers (data not shown), and the elasticity of the SELP shell phase. After methanol treatment, the thickness of both specimens decreased and the elongation at break slightly increased due to the β-sheet crystal structure in SF and the silk domain in the SELPs, which caused contraction and higher packing density of the fiber mats. On the other hand, although tensile strength of SF and SF-SELP mats improved to 2.15 ± 0.32 MPa and 4.81 ± 0.35 MPa, respectively, the Young’s modulus of the SF-SELP core-shell mats was almost the same, and the elongation at break was 5.20% ± 0.57%. These results suggested that the SELPs reduce the brittleness of SF mats whose elongation at break was only1.38% ± 0.22%.

## 4. Conclusions

The recombinant protein polymer SELP and SF aqueous solutions were used to produce SF-SELP core-shell structured nanofibers by coaxial electrospinning. SF solution with high viscosity and SELP solution with low viscosity were utilized as core and shell materials to cospin, respectively. The effects of processing parameters on morphology and diameters of electrospun SF-SELP nanofibers were studied and the SF core solution served as spinning aid to prepare uniform nanofibers with average diameter from 301 ± 108 nm to 408 ± 150 nm via adjusting process parameters. The core-shell structure of SF-SELP nanofibers was confirmed via fluorescence imaging and TEM. The as-spun SF-SELP nanofiber mats were treated with methanol vapor to make fiber mats insoluble in water based on conversion of the secondary structures from random coils to β-sheets. The mechanical testing of nanofiber mats showed that SF-SELP core-shell structured nanofibers had good flexibility with elongation at break of 5.20% ± 0.57% after methanol treatment, compared with SF nanofibers with elongation at break of 1.38% ± 0.22%. SF-SELP core-shell structured nanofibers should provide interesting utility in biomaterial systems and in the biomedical field such as for drug delivery, due to the functional layer on the surface of the SF fibers.

## Figures and Tables

**Table 1:** Process parameters and statistical analysis of nanofiber diameters obtained from the field emission scanning electron microscopy (FE-SEM) images (sample size *N* = 100).

Samples in Figure	Concentration %		Flow Rate mL/h		Applied Voltage kV	Working Distance cm	Mean Diameter nm	Standard Deviation ±nm
	Core	Shell	Inner	Outer				
Figure 3a	26	15	0.4	0.17	20	12	-	-
Figure 3b	29	15	0.4	0.17	20	12	301	108
Figure 3c	32	15	0.4	0.17	20	12	-	-
Figure 4a	29	12	0.4	0.17	20	10	373	129
Figure 4b	29	13.5	0.4	0.17	20	10	406	151
Figure 4c	29	15	0.4	0.17	20	10	361	98

**Table 2:** Process parameters and statistical analysis of nanofiber diameters obtained from the FE-SEM images (sample size *N* = 100).

Samples in Figure	Concentration %		Flow Rate mL/h		Applied Voltage kV	Working Distance cm	Mean Diameter nm	Standard Deviation ±nm
	Core	Shell	Inner	Outer				
Figure 5(A1)	29	12	0.4	0.17	20	12	370	121
Figure 5(A2)	29	12	0.6	0.17	20	12	408	150
Figure 5(A3)	29	12	0.8	0.17	20	12	474	162
Figure 5(B1)	29	12	0.6	0.07	20	12	712	215
Figure 5(B2)	29	12	0.6	0.17	20	12	408	150
Figure 5(B3)	29	12	0.6	0.34	20	12	-	-
Figure 5(C1)	29	12	0.6	0.17	20	12	408	150
Figure 5(C2)	29	12	0.6	0.17	25	12	-	-
Figure 5(C3)	29	12	0.6	0.17	30	12	-	-

**Table 3:** The average thickness, Young’s moduli, tensile strength and elongation at break of SF fibers mats and SF-SELP core-shell fibers mats (sample size *N* = 10).

Samples	Thickness (μm)	Young’s Modulus (MPa)	Tensile Strength (MPa)	Elongation at Break (%)
SF mats	85 ± 8	1.02 ± 0.12	1.00 ± 0.22	1.05 ± 0.15
SF-SELP mats	80 ± 6	1.30 ± 0.35	1.72 ± 0.24	4.70 ± 0.31
SF mats (MeOH)	50 ± 5	2.16 ± 0.17	2.15 ± 0.32	1.38 ± 0.22
SF-SELP mats (MeOH)	45 ± 4	1.32 ± 0.52	4.81 ± 0.35	5.20 ± 0.57
